# Health Information Technology–Related Loss of Central Surveillance Data in a Heart Intensive Care Unit: Multi-Framework Case Report

**DOI:** 10.2196/92560

**Published:** 2026-04-09

**Authors:** Md Shafiqur Rahman Jabin

**Affiliations:** 1 Department of Medicine and Optomtery, Linnaeus University, Universitetsplatsen 1, Kalmar, Sweden, 44 07915673612; 2 Faculty of Health and Social Care, University of Bradford, Bradford, England, United Kingdom

**Keywords:** safety incident analysis, clinical information systems, monitoring system downtime, system availability failure, technology-induced error, incident classification, resilience engineering, safety-critical systems, system recovery processes, intensive care informatics

## Abstract

**Background:**

Centralized electronic surveillance systems are widely used in intensive care settings to support continuous physiological monitoring and patient safety. Failures in health information technology (HIT) infrastructure can disrupt workflows, reduce situational awareness, and create latent risks for serious harm. Understanding such events requires analytic approaches that go beyond single-classification frameworks.

**Objective:**

This study aimed to classify and analyze an HIT-related incident that involved loss of central surveillance data in a heart intensive care unit using multiple complementary patient safety and human factors frameworks.

**Methods:**

This study is a qualitative case report analysis of an incident in which a central surveillance system intermittently lost server connectivity, resulting in unavailability and loss of monitoring data. The narrative was derived from an incident report and supporting documentation and was translated and linguistically adapted for publication. The incident was independently classified using 5 frameworks: the International Classification for Patient Safety (ICPS), the Health Information Technology Classification System (HIT-CS), Systems Engineering Initiative for Patient Safety (SEIPS) 2.0, the sociotechnical model by Sittig and Singh, and the Human Factors Analysis and Classification System for health care (HFACS-Healthcare). Findings were synthesized across frameworks.

**Results:**

All 5 frameworks characterized the event as an HIT-driven system failure involving information unavailability, delayed detection, and multi-patient impact. The HIT-CS identified a technical failure in system availability and recovery. The ICPS classified the event as a documentation or information incident with potential for severe harm. The SEIPS 2.0 and sociotechnical models highlighted disruptions to monitoring tasks and the organization's reliance on IT intervention. The HFACS-Healthcare attributed the event primarily to organizational influences and preconditions for unsafe acts, with no frontline unsafe acts identified. Convergence across frameworks emphasized system-level contributors.

**Conclusions:**

HIT-related monitoring failures in high-acuity settings are best understood as sociotechnical system events rather than isolated technical faults or individual errors. A multi-framework approach provided complementary insights into detection, recovery, and governance vulnerabilities, supporting improved learning and resilience in clinical surveillance systems.

## Introduction

Health information technology (HIT) is increasingly embedded in high-acuity clinical environments, where system reliability is critical for patient safety. Centralized electronic surveillance systems are widely used in intensive and cardiac care units to support continuous physiological monitoring, early detection of deterioration, and retrospective review of patient data [1,2]. While such systems offer substantial safety benefits, failures in HIT infrastructure can disrupt clinical workflows, obscure patient status, and create latent risks for severe harm [3,4]. Therefore, understanding and learning from HIT-related incidents remains a priority for patient safety research [5,6].

Incident reporting systems capture a wide range of adverse events and near-misses, but single-classification approaches often fail to fully capture the complexity of HIT-related failures [6,7]. To address this limitation, several complementary classification frameworks have been developed, each emphasizing different aspects of safety incidents. The World Health Organization’s International Classification for Patient Safety (ICPS) provides a standardized, high-level taxonomy for categorizing incident types, patient outcomes, and contributing factors, supporting comparability across settings and jurisdictions [8]. However, the ICPS does not explicitly focus on the mechanisms underlying HIT failures [9].

To more precisely characterize HIT-related safety events, Magrabi, Jabin, and colleagues developed the Health Information Technology Classification System (HIT-CS), which focuses on information flow, technical failure modes, and outcome manifestations specific to HIT [6,10,11]. The HIT-CS enables detailed identification of where and how HIT failures arise, such as system unavailability, data loss, or delayed detection, and has been widely used in analyses of technology-induced errors [12-14]. Previous research has highlighted limitations in existing incident reporting structures for HIT-related events and emphasized the need for refined classification systems to support meaningful learning and governance [15]. Strengthening classification frameworks is particularly important in high-acuity environments where system-level failures may have multi-patient implications.

In parallel, human factors and systems engineering frameworks offer additional perspectives on how technology interacts with people, tasks, and organizations. The Systems Engineering Initiative for Patient Safety (SEIPS) 2.0 model conceptualizes patient safety as an emergent property of the work system, encompassing interactions among technology, tasks, people, organizational structures, environments, and processes [16]. SEIPS supports the analysis of how HIT failures disrupt clinical work and contribute to unsafe conditions rather than focusing solely on technical faults [17].

Similarly, the sociotechnical model proposed by Sittig and Singh [18,19] emphasizes that HIT safety incidents arise from interdependencies among technical infrastructure, clinical content, human-computer interfaces, workflows, organizational policies, external pressures, and system monitoring. This framework is particularly useful for identifying latent conditions and design vulnerabilities that may not be apparent when incidents are examined through a single disciplinary lens [20].

Finally, human factors classification systems such as the Human Factors Analysis and Classification System (HFACS) for health care (HFACS-Healthcare) provide a structured approach to examine human, supervisory, and organizational contributors to safety events [21]. HFACS-Healthcare allows differentiation between frontline actions, preconditions for unsafe acts, supervisory factors, and organizational influences and is especially valuable for demonstrating when incidents are driven by system-level factors rather than individual error [22,23].

This case report describes an HIT-related incident involving loss of central patient surveillance data in a heart intensive care unit. To provide a comprehensive and methodologically rigorous analysis, the incident was classified using 5 complementary frameworks: ICPS, HIT-CS, SEIPS 2.0, the Sittig and Singh sociotechnical model, and HFACS-Healthcare. By applying multiple classification systems to a single incident, this study aimed to illustrate how different frameworks contribute distinct and complementary insights into HIT-related patient safety risks and to support more comprehensive learning from HIT failures.

## Methods

### Study Design

This study is a qualitative case report that describes and analyzes an HIT-related patient safety incident that occurred in a heart intensive care unit. A single-incident case design was chosen to enable an in-depth examination of technical, organizational, and human factors contributing to the event. Case reports are well suited for studying rare or complex HIT failures and for generating transferable safety insights when experimental or quantitative approaches are not feasible [1,3,12,24].

### Ethical Considerations

This study involved secondary analysis of a fully deidentified incident report obtained from a national incident reporting repository. The data contained no patient identifiers or confidential information. Ethical advice was obtained from the Ethical Advisory Board in southeast Sweden (Dnr 934‐2023). Based on the retrospective analysis of anonymized quality and safety data, formal ethical review and informed consent were not required under applicable regulations (the Swedish Ethical Review Act 2003:460) [25].

### Data Source and Incident Selection

The incident analyzed in this study was obtained from a national incident reporting repository that captures medical device–related and HIT-related events reported by health care professionals and clinical engineering staff. The incident reports in the repository typically include structured fields and free-text narratives describing the incident, the results of any internal investigation, and measures taken in response [26-28]. A single incident was selected for analysis on the basis of the following criteria:

- involvement of a medical device with embedded software,
- manifestation of the incident during active clinical care,
- relevance to HIT-mediated information loss, and
- availability of sufficient narrative detail to support qualitative analysis.

The incident report included 3 core narrative components: an incident description, a summary of investigation findings, and a summary of measures. These components formed the primary data for analysis (Textbox 1). The incident description was translated and linguistically adapted from the original incident report to improve clarity and readability, without altering the factual content.

Textbox 1. **Description of a health information technology–related incident involving loss of central patient surveillance data in a heart intensive care unit, including incident description, investigation findings, and implemented or proposed safety measures.**

**Incident description**

On January 18, 2026, the central surveillance system in a heart intensive care unit lost connection with its server on 2 occasions, around 11 AM and 1 PM. During these periods, real-time patient monitoring data were unavailable and not stored. The issue was not detected immediately and became apparent at 4 PM when staff attempted to review recorded surveillance data. A trained superuser attempted to restart the system using previously distributed instructions, but the restart command did not respond, and the system interface subsequently froze. The IT department was contacted and performed a restart of the main computer.

**Summary of investigation**

The investigation identified a failure in the health information technology infrastructure, specifically the loss of server connectivity affecting the central surveillance system. The incident was compounded by delayed detection, as there were no real-time alerts indicating loss of data capture or system disconnection. Restart procedures that relied on local user action were ineffective, and the system entered an unresponsive state requiring IT intervention. The event highlighted dependencies between clinical monitoring, server availability, and recovery procedures, as well as limitations in system resilience and monitoring transparency.

In a heart intensive care unit, continuous centralized surveillance supports early detection of life-threatening arrhythmias, hemodynamic instability, and acute clinical deterioration. Therefore, loss of real-time and stored monitoring data creates a risk that critical physiological changes may go unnoticed or recognition may be delayed, particularly in settings where centralized monitoring complements bedside observation. Although no confirmed patient harm was identified in this case, the interruption of surveillance constituted a significant patient safety vulnerability.

**Summary of measures**

Immediate measures included restarting the central surveillance system with assistance from the IT department to restore functionality. Planned and recommended measures focused on improving system reliability and patient safety, including implementation of automated alerts for loss of server connectivity, clarification and testing of restart and recovery procedures, improved collaboration between clinical staff and IT services, and evaluation of redundancy or fail-safe mechanisms to prevent loss of monitoring data during system downtime.

### Data Preparation and Deidentification

The original incident report was written in a language other than English and contained informal clinical and technical expressions typical of voluntary incident reporting. The report was translated into English using a direct linguistic translation approach focused on preserving semantic accuracy. No interpretive synthesis or restructuring of factual content was performed. Following translation, minor language editing was undertaken to improve clarity, including grammatical corrections and standardization of terminology, without altering the original meaning or sequence of events. The translated version was reviewed by a clinical engineer experienced in medical device incident management to ensure technical accuracy and contextual fidelity.

To ensure confidentiality and compliance with ethical and publication standards, all potentially identifying information, including names of individuals, institutions, manufacturers, locations, dates, and device identifiers, was removed prior to analysis. The final narrative was reviewed to confirm that it remained fully deidentified while retaining sufficient detail for analytical purposes [29].

### Analytical Approach

To provide a comprehensive and systems-oriented analysis, the incident was classified using five complementary patient safety and human factors frameworks: (1) ICPS, (2) HIT-CS, (3) the SEIPS 2.0 model, (4) the sociotechnical model for HIT safety proposed by Sittig and Singh, and (5) HFACS-Healthcare.

Each framework was applied independently to the same incident narrative to capture different dimensions of the event, including incident type and outcome (ICPS), HIT-specific failure mechanisms (HIT-CS), work system interactions (SEIPS 2.0), sociotechnical dependencies (Sittig and Singh sociotechnical model), and human and organizational contributors (HFACS-Healthcare).

### International Classification for Patient Safety

The ICPS was used to classify the incident at a high level, including incident type, detection, patient outcome, and degree of harm. It provides an internationally standardized taxonomy for patient safety incidents and supports comparison across settings and reporting systems [8]. The framework was applied descriptively to characterize the incident without attributing causality.

### Health Information Technology Classification System

The HIT-CS, developed by Magrabi, Jabin, and colleagues, was used to classify the HIT-specific characteristics of the incident, including the primary issue type, secondary technical issues, outcome manifestation, workflow location, and impact scale [6,10,11]. The HIT-CS focuses on information flow and technical failure modes and is particularly suited for identifying technology-induced risks and system unavailability events [30,31].

### SEIPS 2.0 Work System Analysis

The SEIPS 2.0 framework was applied to examine how the incident affected and emerged from interactions among technology, tasks, people, organizational structures, environment, and processes [16]. This analysis emphasized that the surveillance system failure disrupted continuous monitoring and retrospective data review and that recovery depended on organizational coordination with IT services.

### Sociotechnical Classification

The sociotechnical model proposed by Sittig and Singh [18,19] was used to analyze interdependencies among technical infrastructure, clinical content, human-computer interfaces, workflows, organizational policies, and system monitoring. This framework was applied to identify latent conditions such as lack of system status visibility and centralized architecture that contributed to delayed detection and multi-patient impact [4,18,20].

### HFACS-Healthcare Classification

HFACS-Healthcare was applied to examine human and organizational contributors to the incident across 4 levels: unsafe acts, preconditions for unsafe acts, unsafe supervision, and organizational influences [21,32]. This framework was used to distinguish between frontline performance and system-level factors and to assess whether human error contributed to the incident. HFACS-Healthcare was applied analytically rather than punitively, consistent with its use in health care safety research [22,23,33].

**Figure 1.**
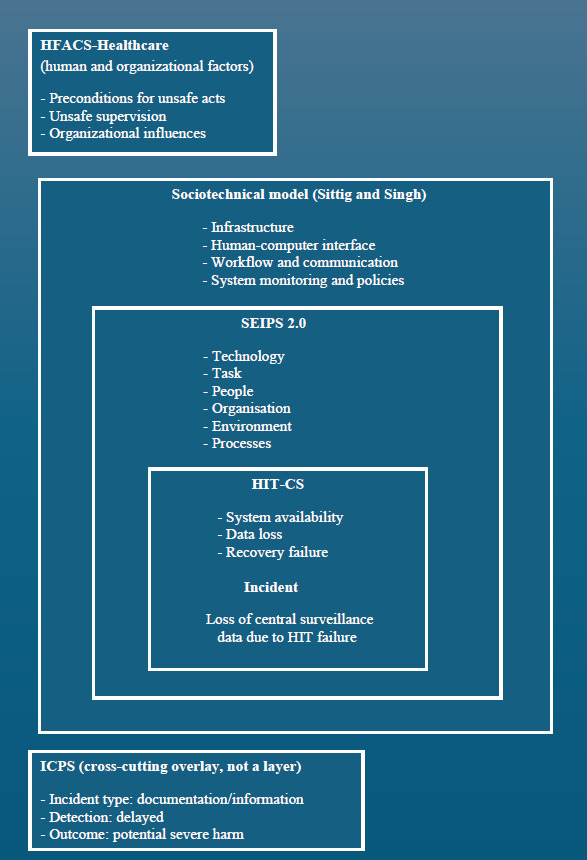
Multi-framework conceptual mapping of the HIT-related incident. HFACS, Human Factors Analysis and Classification System; HIT, health information technology; HIT-CS, Health Information Technology Classification System; ICPS, International Classification for Patient Safety; SEIPS, Systems Engineering Initiative for Patient Safety.

Figure 1 illustrates the conceptual relationship among the 5 classification frameworks applied in this study and their complementary roles in characterizing the incident.

## Results

### Overview of Incident Classification

The incident was analyzed using 5 complementary classification frameworks to capture its patient safety, technical, sociotechnical, and human factors dimensions. This section summarizes the classification results according to each framework. Together, these analyses demonstrated that the incident was primarily driven by HIT system failures and latent organizational and design factors rather than by frontline human error.

#### ICPS Classification

Using the ICPS, the incident was primarily classified as a documentation/information incident, with unavailable and lost patient monitoring data as the central safety concern. A secondary classification under medical device/equipment reflected the role of a centralized electronic surveillance system in clinical monitoring. Detection was classified as delayed, as the system failure was identified retrospectively during data review rather than at the time of occurrence. No confirmed patient harm was identified; however, the incident was classified as having potential for severe harm due to the risk of missed or delayed recognition of life-threatening cardiac events. Table 1 presents the detailed ICPS classification, including incident type, detection, degree of harm, and contributing factors.

**Table 1.** Classification of the incident according to the International Classification for Patient Safety (ICPS).

ICPS domain and classification	Description applied to this incident
Incident type (primary)	
Documentation/information	Unavailable and lost patient monitoring data due to a loss of connection between the central surveillance system and the server
Incident type (secondary)	
Medical device/equipment (health information technology)	Failure of a central electronic surveillance system supporting physiological monitoring
Patient outcome	
No harm (outcome unknown)	No confirmed patient injury was identified; patient impact could not be fully assessed due to missing data.
Degree of harm	
No harm/potential harm	Potential for severe harm due to the risk of missed or delayed detection of life-threatening cardiac events
Detection	
Delayed detection	The incident was identified retrospectively when staff attempted to review stored monitoring data.
Contributing factors/hazards	
Organizational/system factors	Lack of automated alerts for system disconnection; reliance on a centralized monitoring infrastructure
Technical factors	Server connectivity failure; ineffective restart function; system freeze requiring IT intervention
Mitigating factors	
Human factors	Presence of a trained superuser who attempted recovery procedures
Ameliorating actions	
System-level response	Restart of the main system by the IT department and review of system recovery procedures

No confirmed patient harm was identified; however, the incident was classified as having potential for severe harm due to the risk of missed or delayed recognition of life-threatening cardiac events. In high-acuity cardiac care, even short periods of absent surveillance may increase the risk of undetected arrhythmias or delayed response to clinical deterioration.

#### HIT-CS Classification

Using the HIT-CS, the incident was classified as a technical HIT failure that affected system availability and connectivity. The primary issue type was classified as a software/hardware (technical) failure, specifically loss of system availability due to server disconnection. A secondary technical issue involved a system recovery failure: user-initiated restart commands did not function, and the system became unresponsive. The outcome manifestation was classified as information unavailable/data loss, reflecting both an interruption of real-time monitoring and a permanent loss of stored surveillance data.

The HIT-CS analysis also highlighted the workflow location of the failure within continuous physiological surveillance and retrospective data review, as well as a multi-patient scale characteristic, given that the central surveillance system supported simultaneous monitoring of multiple patients. Similar multi-patient propagation patterns following HIT failures have been reported in Swedish incident analyses, where system-level disruptions affected care management across multiple patients simultaneously [30]. Table 2 summarizes the HIT-CS classification dimensions and their application to the incident.

**Table 2.** Classification of the incident using the Health Information Technology Classification System (HIT-CS) framework.

HIT-CS dimension	HIT-CS category	Specific subtype (per HIT-CS)	How it applies to this incident
Primary issue type	Software/hardware (technical)	System availability/connectivity failure	Loss of connection between the central surveillance system and the server caused intermittent unavailability of real-time and stored patient monitoring data.
Secondary technical issue	Software/hardware (technical)	System recovery/restart failure	Restart commands initiated via the system interface did not execute, and the system entered an unresponsive (frozen) state requiring IT intervention.
Outcome manifestation	Information (machine/output)	Information unavailable/data loss	Patient monitoring data were not displayed in real time or stored, resulting in permanent gaps in surveillance records.
Optional contributing factor	Information (use/human)	Delayed detection/delayed monitoring	The system failure was not immediately apparent and was detected retrospectively when staff attempted to review recorded data several hours later.
Workflow location (contextual)	HIT-mediated^a^ clinical monitoring workflow	Continuous physiological surveillance/data review	The failure occurred at the stage where the central surveillance system transmits, displays, and archives physiological data.
Scale characteristic	HIT system behavior	Multi-patient impact	The central surveillance system supports the simultaneous monitoring of multiple patients; therefore, the loss of functionality affected all monitored patients during downtime.

a HIT: health information technology.

#### SEIPS 2.0 Classification

Using the SEIPS 2.0 framework, the incident was characterized as a disruption of the clinical work system arising from interactions among technology, tasks, people, organization, environment, and processes. The technology domain captured the central surveillance system’s loss of reliability. The task analysis showed that neither continuous monitoring nor retrospective data review could be performed as intended. The people domain indicated appropriate user actions, with staff following established restart instructions without success.

At the organizational level, system recovery depended on escalation to IT services, highlighting the reliance on centralized technical support. The environment domain reflected dependency on a single central surveillance workspace, while the processes domain identified delayed incident detection and reactive response as key characteristics. The outcome domain captured the potential for patient harm due to unavailable monitoring data. The SEIPS 2.0 classification is presented in Table 3.

**Table 3.** Classification of the incident according to Systems Engineering Initiative for Patient Safety (SEIPS) 2.0.

SEIPS 2.0 domain	Classification	How it applies to this incident
Technology and tools	Central surveillance system reliability	Loss of server connectivity and failure of system restart functionality rendered the monitoring system unavailable.
Tasks	Continuous physiological monitoring and data review	Real-time surveillance and retrospective review of patient monitoring data could not be performed as intended.
People	Clinical staff and superusers	Staff appropriately followed established restart instructions but were unable to recover system functionality.
Organization	IT support and escalation pathways	Resolution depended on the IT department’s intervention, highlighting the reliance on centralized technical support.
Environment	Central surveillance workspace	The monitoring environment depended on a single central system, amplifying the impact of system failure.
Processes	Incident detection and response	The lack of automated alerts led to delayed detection and a reactive response after a retrospective data review.
Outcomes	Potential patient harm	Risk of missed or delayed detection of life-threatening cardiac events due to unavailable monitoring data

#### Sociotechnical Classification (Sittig and Singh Model)

The sociotechnical analysis identified multiple interacting system components contributing to the incident. Hardware and software infrastructure failures resulted in system unavailability and data loss, while deficiencies in the human-computer interface limited users’ ability to understand the system state or recover functionality. The workflow and communication dimensions revealed delayed awareness and escalation due to the absence of system status alerts.

At the organizational level, recovery procedures depended on IT intervention, and the system lacked redundancy or fail-safe mechanisms to maintain monitoring during outages. The system measurement and monitoring dimension highlighted the absence of automated alerts for loss of connectivity or for data capture failures. These interacting sociotechnical factors created latent conditions that amplified the impact of the technical failure and increased patient safety risk. Table 4 presents a comprehensive sociotechnical classification of the incident.

**Table 4.** Sociotechnical classification of the incident (Sittig and Singh model).

Sociotechnical dimension	Classification	How it applies to this incident
Hardware and software computing infrastructure	System connectivity and availability failure	Loss of connection between the central surveillance system and the server caused intermittent system unavailability and data loss.
Clinical content	Availability and completeness of monitoring data	Physiological monitoring data were unavailable in real time and not stored, resulting in gaps in clinical information.
Human-computer interface	Lack of system feedback and controllability	Restart commands produced no visible response, and the system interface became unresponsive, preventing user-led recovery.
People	Roles, training, and system use	A trained superuser followed established restart instructions appropriately but was unable to restore functionality.
Workflow and communication	Monitoring, review, and escalation processes	The absence of real-time alerts delayed detection; escalation occurred only after a retrospective review of missing data.
Internal organizational policies and procedures	System recovery and downtime procedures	Recovery depended on contacting IT services, indicating limited local recovery capability and unclear resilience to downtime.
External rules, regulations, and pressures	Patient safety and monitoring requirements	Continuous cardiac monitoring is a regulatory and clinical safety expectation; system failure undermined this requirement.
System measurement and monitoring	System health visibility and alerting	No automated alerts indicated loss of server connectivity or data capture failure, contributing to delayed detection.
System configuration and upgrades	Configuration resilience	Centralized system architecture lacked redundancy or fail-safe mechanisms to maintain monitoring during server outages.
Outcomes and consequences	Potential patient harm	Risk of missed or delayed detection of life-threatening cardiac events affecting multiple patients simultaneously.

#### HFACS-Healthcare Classification

The HFACS-Healthcare analysis demonstrated that the incident was not associated with unsafe acts by frontline staff. At the level of preconditions for unsafe acts, technological environment factors such as poor system feedback and centralized system dependency reduced situational awareness and limited recovery options. At the unsafe supervision level, reliance on manual detection and the absence of real-time system oversight contributed to the delayed recognition of the failure.

At the organizational level, resource management and system design decisions, including limited redundancy and reliance on centralized IT support for recovery, were identified as key contributors. Overall, the HFACS-Healthcare analysis indicated that the incident arose primarily from latent system and organizational factors rather than from individual human error. The HFACS-Healthcare classification is summarized in Table 5.

**Table 5.** Classification of the incident according to the Human Factors Analysis and Classification System (HFACS)-Healthcare.

HFACS-Healthcare level and category	Classification	How it applies to this incident
Level 1: unsafe acts		
Errors	No unsafe acts identified	Clinical staff and the superuser acted appropriately and followed established restart procedures; no misuse or deviation was identified.
Level 2: preconditions for unsafe acts		
Technological environment	Poor system feedback and controllability	The surveillance system provided no feedback when restart commands were issued and became unresponsive, limiting users’ ability to understand or manage the system state.
Physical/technological environment	Centralized system dependency	A single central surveillance system supported multiple patients, thereby increasing the impact when it failed.
Cognitive factors	Reduced situational awareness	Staff were unaware of the system failure until retrospective review, indicating diminished awareness of system status rather than clinical oversight.
Level 3: unsafe supervision		
Inadequate supervision	Insufficient monitoring of system health	There was no active oversight mechanism or real-time alerting to detect loss of server connectivity or data capture.
Planned inappropriate operations	Reliance on manual detection	Continued operation without automated system status alerts assumed that failures would be noticed through routine use.
Level 4: organizational influences		
Resource management	Limited redundancy and resilience	The surveillance architecture lacked redundancy or fail-safe mechanisms to maintain monitoring during server outages.
Organizational climate	Separation between the clinical and IT domains	Recovery depended on IT intervention, reflecting organizational boundaries that limited frontline recovery capacity.
Organizational processes	Downtime and recovery procedures	Restart instructions existed but were insufficient for nonresponsive system states, requiring escalation beyond clinical control.

### Cross-Framework Synthesis

Across all 5 classification frameworks, the incident was consistently characterized as an HIT-driven system failure with delayed detection, multi-patient impact, and potential for severe harm. While each framework emphasized different aspects of the incident, all converged on the absence of frontline human error and the presence of latent technical, organizational, and design vulnerabilities. The combined use of the ICPS, HIT-CS, SEIPS 2.0, sociotechnical, and HFACS-Healthcare frameworks provided a comprehensive and complementary understanding of the incident and its patient safety implications. To facilitate comparison across frameworks and to synthesize key findings, Table 6 presents an alignment of major incident characteristics as identified by the ICPS, HIT-CS, SEIPS 2.0, Sittig and Singh sociotechnical model, and HFACS-Healthcare. Despite differences in terminology and analytical focus, the frameworks showed strong convergence in identifying the incident as a technology-driven failure with delayed detection, multi-patient impact, and predominant system-level and organizational contributors rather than frontline human error.

**Table 6.** Cross-framework alignment of key findings from the classification of a health information technology–related incident using the ICPS^a^, HIT-CS^b^, SEIPS 2.0^c^, Sittig and Singh sociotechnical model, and HFACS^d^-Healthcare, highlighting convergent and complementary perspectives on incident nature, detection, human contribution, organizational factors, and impact.

Analytic theme	ICPS	HIT-CS	SEIPS 2.0	Sociotechnical (Sittig and Singh) model	HFACS-Healthcare
Primary incident nature	Documentation/information	System availability failure	Technology domain	Infrastructure failure	Preconditions (technological environment)
Detection	Delayed detection	Delayed detection	Process failure	Lack of system monitoring	Unsafe supervision
Human contribution	Not specified	Appropriate use	People domain	Human-computer interface	No unsafe acts
Organizational factors	Contributing factors	Workflow location	Organization	Policies and procedures	Organizational influences
Scale of impact	Potential severe harm	Multi-patient impact	Outcome	Centralized architecture	Resource management
Recovery	Mitigation actions	Recovery failure	Processes	IT dependency	Supervisory dependence

a ICPS: International Classification for Patient Safety.

b HIT-CS: Health Information Technology Classification System.

c SEIPS: Systems Engineering Initiative for Patient Safety.

d HFACS: Human Factors Analysis and Classification System.

## Discussion

### Principal Findings

This case report demonstrates that an HIT failure in a high-acuity clinical setting can pose a substantial patient safety risk despite appropriate frontline actions. Across all 5 applied classification frameworks—that is, ICPS, HIT-CS, SEIPS 2.0, the Sittig and Singh sociotechnical model, and HFACS-Healthcare—the incident was consistently characterized as a system-driven failure involving loss of monitoring information, delayed detection, and multi-patient impact. The absence of frontline human error identified across frameworks aligns with prior research showing that many HIT-related safety events arise from latent system conditions rather than individual mistakes [3,4,11,19].

Importantly, the incident should not be interpreted as a benign technical malfunction. In high-acuity cardiac settings, centralized surveillance serves as a safety layer to detect arrhythmias and sudden physiological deterioration. Interruption of this layer introduces a window of vulnerability during which time-critical events may be missed or responses delayed, underscoring the clinical seriousness of HIT availability failures. Incident report analyses have demonstrated that software-related challenges frequently reflect latent system conditions rather than isolated user errors, reinforcing the importance of sociotechnical interpretation of HIT failures [34].

The alignment of findings across frameworks (Table 6) illustrates how different taxonomies emphasize complementary dimensions of the same event. The ICPS provided a standardized patient safety classification [7,8], the HIT-CS enabled precise characterization of HIT-specific failure mechanisms [9,10], and the SEIPS, sociotechnical, and HFACS-Healthcare frameworks highlighted how interactions among technology, workflows, supervision, and organizational context shaped the emergence and impact of incidents [13,15,19].

### Methodological Contribution

This study contributes methodologically by demonstrating the value of multi-framework incident classification. Prior work has noted that reliance on a single taxonomy may obscure important aspects of HIT-related risk [6,11]. In this case, the HIT-CS clarified technical failure modes; the SEIPS and sociotechnical models contextualized the disruption to clinical work; and the HFACS-Healthcare provided a structured explanation of how organizational and supervisory factors contributed to the disruption, without attributing blame to frontline staff [18,19].

The integrative alignment table (Table 6) supports synthesis across frameworks and reduces interpretive fragmentation. Similar alignment approaches have been recommended to strengthen learning from complex safety events and to improve translation of analytic findings into practice [22,24].

### Technological Failures as System-Level Safety Events

Failure of centralized HIT should be interpreted through the lens of systemic safety governance, rather than isolated technical faults. Complex care, such as extracorporeal treatments, requires structured communication, protocols, and organizational support to mitigate clinical risk. Nalesso et al [35] emphasize the importance of proactive and reactive safety management in critical care settings, including multidisciplinary risk assessment, procedural checklists, and safety culture tools to reduce clinical incidents in high-risk technologies.

High-risk technologies in critical care demand governance approaches that extend beyond device functionality to include organizational coordination, communication, and monitoring infrastructure. In complex therapies such as extracorporeal blood purification, safety governance includes structured protocols, feedback loops, and systematic risk analysis to prevent latent failures that could lead to patient harm [18,35]. Digital incident reporting systems themselves require quality governance to ensure meaningful learning from HIT failures. Prior work has demonstrated variability in documentation quality and analytic depth within digital reporting systems, underscoring the need for structured governance frameworks [28].

### Remote Monitoring and Alerting: Benefits and Limitations

Remote monitoring and device control can improve patient care and staff safety, but such systems must be interpreted in context with cognitive load and alert management. Garzotto et al [36] describe how remote control of medical devices reduced risk and unnecessary interventions in the intensive care unit during the COVID-19 pandemic, highlighting operational benefits and the potential for remote surveillance to support safety in critical settings [37].

Remote monitoring and control of medical devices can reduce direct exposure risks and streamline clinical workflows, particularly during infectious outbreaks. Garzotto et al [36] reported that remote device control decreased unnecessary interventions and supported patient safety in intensive care environments [37,38]. However, as with all automated systems, trade-offs such as increased alert burden and clinician cognitive load must be considered to ensure that alerting improves situational awareness without inducing fatigue [37,38].

### Telemedicine and Remote Surveillance Concepts

Although not specific to intensive care unit central surveillance, telemedicine and remote monitoring paradigms offer conceptual bridges for understanding distributed monitoring and alerting across care settings. Ricci and Ronco [39] describe telemedicine support for peritoneal dialysis, illustrating how continuous remote surveillance of treatment data provides clinical oversight and improves outcomes in chronic care. These principles parallel the reliability of centralized surveillance; both involve automated data capture, transmission, and interpretation to support timely clinical decisions [35,40,41].

Concepts from telemedicine and continuous remote monitoring provide relevant frameworks for ensuring the reliability of centralized surveillance. Remote monitoring of chronic therapies such as peritoneal dialysis involves continuous data transmission and clinician oversight, improving the detection of therapy deviations and enabling timely intervention [35,41,42].

### IT and Safety in Critical Care Therapies

Early literature emphasizes that IT in critical care therapies (such as continuous renal replacement therapy) impacts patient safety, monitoring, and documentation. Ricci and Ronco [39] underscored that IT contributes to practice variation, patient assessment, monitoring, and documentation in critical care, highlighting the potential for technology to influence safety outcomes when integrated with governance and quality systems [4].

IT implementation in critical care therapies can enhance patient assessment, documentation, and therapeutic monitoring, but it also introduces new failure modes that must be governed within broader safety and quality infrastructures [4,37,39].

### Implications for Clinical Practice

#### Early Detection of HIT Failures

Delayed detection emerged as a dominant theme across all classification approaches. Similar delays in recognizing HIT failures have been described in prior studies, particularly in settings that rely on retrospective review rather than real-time system monitoring [11,28]. These findings underscore the importance of implementing automated system status alerts and monitoring dashboards to support timely detection of connectivity loss, data capture failure, or system degradation [16,26].

From a practical perspective, health care organizations should consider the following:

- Deployment of automated system status alerts distinct from physiological alarms
- Real-time monitoring dashboards accessible to both clinical units and IT services
- Clear escalation pathways triggered by connectivity or data integrity failures

However, implementation of automated alerts must be carefully designed to avoid unintended consequences such as alert fatigue and increased cognitive load among clinical staff. Excessive or nonspecific alerts may desensitize users and reduce responsiveness, particularly in intensive care environments already characterized by high alarm burden [1,2]. Therefore, system status alerts for surveillance failures should be prioritized, clearly distinguishable from routine physiological alarms, and integrated into existing workflows to balance early detection with usability and clinician attention.

#### Managing Operational Vulnerabilities in HIT Environments

Similar operational disruptions following software modifications, including security patching, have been documented in health care settings, illustrating how even routine IT maintenance may introduce unintended workflow vulnerabilities [27]. This reinforces the need for structured change management processes and proactive risk assessment before implementing system updates in high-acuity environments.

Evidence from quality improvement interventions in radiology demonstrates that structured system redesign and multidisciplinary engagement are more effective than isolated procedural adjustments, further supporting system-level corrective strategies for HIT-related failures [43,44]. Accordingly, mitigation efforts should emphasize the following:

- Multidisciplinary planning for HIT upgrades and maintenance
- Prospective hazard analysis before system changes
- Integration of IT governance with clinical safety leadership

#### Recovery Capacity and Local Resilience

The case also highlights limitations in frontline recovery capacity. Despite appropriate use by trained superusers, recovery required IT intervention, reflecting a lack of graduated recovery mechanisms at the clinical unit level. Human factors research emphasizes that resilient systems should support adaptation and recovery close to the point of failure, rather than relying solely on centralized escalation [22,23].

Designing HIT systems that provide meaningful feedback during recovery attempts and clearly guide escalation pathways may reduce downtime and associated patient risk. Practical measures may include the following:

- Transparent system status indicators during restart attempts
- Tiered recovery protocols with defined thresholds for escalation
- Simulation-based training for downtime scenarios

#### Governance, Redundancy, and Architectural Risk

From an organizational perspective, the incident illustrates the safety implications of centralized HIT architectures without redundancy. Central surveillance systems offer efficiency but can amplify harm when failures occur, a phenomenon previously described in analyses of large-scale HIT incidents [26,28]. Explicit assessment of single points of failure and investment in redundancy or fail-safe monitoring strategies are therefore critical in intensive care environments.

Broader analyses of digital technology implementation during crisis conditions have demonstrated that technological reliability, equity, and organizational readiness are critical determinants of safe digital transformation in health care systems [45,46]. These findings support the need for the following:

- System-level governance structures overseeing HIT safety
- Regular auditing of architectural vulnerabilities
- Investment in redundancy for high-acuity monitoring systems
- Integration of clinical, technical, and leadership oversight

#### Translation of Multi-Framework Findings Into Action

Building on the findings from the multi-framework analysis and the implications outlined above, a set of corrective and preventive strategies were devised to mitigate the risk of similar HIT-related surveillance failures in high-acuity care settings (see Textbox 2). These strategies were developed by synthesizing insights from the ICPS, HIT-CS, SEIPS 2.0, the sociotechnical model of Sittig and Singh, and HFACS-Healthcare, with an emphasis on system-level resilience, early detection, recovery capacity, and organizational coordination rather than on individual performance.

Textbox 2. **Preventive and corrective strategies to mitigate health information technology–related central surveillance failures.**

**System reliability and infrastructure resilience**

- Establish redundancy for central surveillance systems, including failover servers or mirrored systems, to reduce the impact of single points of failure in high-acuity settings.
- Implement automatic buffering or local storage of monitoring data to prevent data loss during temporary server connectivity disruptions.
- Ensure routine stress testing and resilience testing of surveillance systems under peak load and failure scenarios.

**System monitoring, detection, and alerting**

- Implement real-time system health monitoring with automated alerts for loss of server connectivity, data transmission failure, or interruption of data storage.
- Ensure that system status indicators are clearly visible to clinical users, indicating whether monitoring data are actively being captured and stored.
- Configure alerts to notify both clinical units and IT services simultaneously to enable parallel awareness and response.

**Recovery procedures and contingency planning**

- Develop and validate clear, stepwise recovery procedures for surveillance system failures, including criteria for escalation to IT support.
- Design restart and recovery functions that provide explicit user feedback (eg, confirmation messages or error notifications) to avoid ambiguity during recovery attempts.
- Formalize fallback monitoring workflows during downtime, such as increased bedside monitoring or alternative documentation processes, to maintain patient surveillance.

**Human factors and training**

- Provide regular training for clinical staff and designated superusers on recognition of abnormal system behavior, limits of local recovery actions, and early escalation pathways.
- Conduct interdisciplinary simulation or tabletop exercises involving clinical staff and IT personnel to rehearse detection, response, and recovery from HIT failures.
- Reinforce a nonpunitive reporting culture to encourage early reporting of HIT anomalies and near-misses.

**Organizational governance and coordination**

- Clearly define roles and responsibilities between clinical units, IT services, and vendors for surveillance system maintenance, monitoring, and incident response.
- Integrate HIT failure scenarios into organizational risk management and patient safety governance structures.
- Ensure that timely communication mechanisms are in place to inform all affected clinical staff of unexpected system downtimes and recovery status.

**System design and integration**

- Design surveillance systems to align with clinical workflows, minimizing reliance on retrospective data review for detection of system failures.
- Ensure interoperability between bedside monitoring devices and central surveillance systems to support continuity of monitoring during partial system failures.
- Incorporate human-centered design principles to improve system transparency, controllability, and usability in safety-critical situations.

The proposed strategies are grouped into key sociotechnical domains to facilitate practical implementation and learning.

### Strengths and Limitations of the Study

A key strength of this study is the systematic application of 5 established classification frameworks to a single HIT-related incident, enabling a comprehensive and triangulated analysis. This approach aligns with calls for richer sociotechnical analyses of HIT safety events [15,16]. Additionally, focusing on an incident with high potential severity but no confirmed harm supports proactive safety learning, consistent with modern patient safety principles [5,23].

Several limitations should be acknowledged. The analysis is based on a single incident report, limiting generalizability and precluding causal inference. Patient outcomes could not be fully assessed due to missing monitoring data, which constrained the classification of harm under ICPS [7]. Furthermore, while the frameworks used are well established, their application involves interpretive judgment; alternative analysts might emphasize different dimensions. Nevertheless, the strong convergence of findings across frameworks suggests robust analytical results.

In addition, the reconstruction of the event relied primarily on the narrative documented in the incident report; it is possible that relevant contextual factors were not recorded and therefore could not be captured within the analytic frameworks applied.

### Implications for Future Research

Future research should examine whether similar patterns of delayed detection, limited recovery capacity, and organizational dependency are observed across larger samples of HIT-related incidents using comparable multi-framework approaches [11,28]. Comparative studies evaluating how different classification systems influence safety recommendations could further guide methodological choices in HIT safety research.

There is also a need for empirical evaluation of design and governance interventions, such as real-time HIT health monitoring, redundancy strategies, and improved IT-clinical coordination models, to assess their effectiveness in reducing risk in high-acuity settings [16,26,29]. Finally, integrating resilience-oriented concepts such as Safety-II into HIT safety analysis may offer additional insights into how systems can better support adaptive clinical work under failure conditions [22,23].

### Conclusions

This case report demonstrates that HIT-related safety incidents in intensive care settings are best understood as emergent properties of complex sociotechnical systems rather than isolated technical malfunctions or individual errors. The combined application of ICPS, HIT-CS, SEIPS 2.0, the sociotechnical model, and HFACS-Healthcare revealed convergent system-level vulnerabilities involving delayed detection, limited recovery capacity, and architectural and organizational design factors. These findings underscore the importance of governance structures, redundancy planning, and human-centered alerting strategies in high-acuity monitoring environments. Integrative, multi-framework analysis may strengthen learning from HIT failures and support safer design, implementation, and resilience of clinical surveillance systems.

## Abbreviations

HFACS: Human Factors Analysis and Classification System
HFACS-Healthcare: HFACS for health care
HIT: health information technology
HIT-CS: Health Information Technology Classification System
ICPS: International Classification for Patient Safety
SEIPS: Systems Engineering Initiative for Patient Safety
